# Characterization of Macroinvertebrate Communities in the Hyporheic Zone of River Ecosystems Reflects the Pump-Sampling Technique Used

**DOI:** 10.1371/journal.pone.0164372

**Published:** 2016-10-10

**Authors:** Rachel Stubbington, Marie-José Dole-Olivier, Diana M. P. Galassi, John-Paul Hogan, Paul J. Wood

**Affiliations:** 1 School of Science and Technology, Nottingham Trent University, Nottingham, Nottinghamshire, United Kingdom; 2 Université de Lyon, Université Claude Bernard Lyon 1, Villeurbanne Cedex, France; 3 Department of Life, Health and Environmental Sciences, University of L'Aquila, L'Aquila, Italy; 4 Centre for Hydrological and Ecosystem Science, Department of Geography, Loughborough University, Loughborough, Leicestershire, United Kingdom; Stockholm University, SWEDEN

## Abstract

The hyporheic zone of river ecosystems provides a habitat for a diverse macroinvertebrate community that makes a vital contribution to ecosystem functioning and biodiversity. However, effective methods for sampling this community have proved difficult to establish, due to the inaccessibility of subsurface sediments. The aim of this study was to compare the two most common semi-quantitative macroinvertebrate pump-sampling techniques: Bou-Rouch and vacuum-pump sampling. We used both techniques to collect replicate samples in three contrasting temperate-zone streams, in each of two biogeographical regions (Atlantic region, central England, UK; Continental region, southeast France). Results were typically consistent across streams in both regions: Bou-Rouch samples provided significantly higher estimates of taxa richness, macroinvertebrate abundance, and the abundance of all UK and eight of 10 French common taxa. Seven and nine taxa which were rare in Bou-Rouch samples were absent from vacuum-pump samples in the UK and France, respectively; no taxon was repeatedly sampled exclusively by the vacuum pump. Rarefaction curves (rescaled to the number of incidences) and non-parametric richness estimators indicated no significant difference in richness between techniques, highlighting the capture of more individuals as crucial to Bou-Rouch sampling performance. Compared to assemblages in replicate vacuum-pump samples, multivariate analyses indicated greater distinction among Bou-Rouch assemblages from different streams, as well as significantly greater consistency in assemblage composition among replicate Bou-Rouch samples collected in one stream. We recommend Bou-Rouch sampling for most study types, including rapid biomonitoring surveys and studies requiring acquisition of comprehensive taxon lists that include rare taxa. Despite collecting fewer macroinvertebrates, vacuum-pump sampling remains an important option for inexpensive and rapid sample collection.

## Introduction

The hyporheic zone comprises the subsurface sediments beneath a streambed that exchange water with the surface stream and the underlying aquifer [1]. Hyporheic sediments have long been recognized as an important habitat for many invertebrates [2, 3], and research into the ecological functioning of the hyporheic zone and its resident biota has increased considerably in recent decades [1, 4, 5]. Alongside a permanent hyporheic community, many predominantly benthic macroinvertebrates migrate vertically to exploit the subsurface sediments as a nursery that protects juveniles from predation [6, 7] and as a refuge that promotes persistence during disturbance events in surface streams [8–10].

In addition to research-focussed interest in hyporheic invertebrates and the ecosystem functions they perform [11, 12], this fauna has been proposed as a biomonitor of ecosystem health, for example in temporary streams [13, 14] and in response to metal pollution [15] and eutrophication [16]. The stygofaunal (groundwater-associated) component of hyporheic communities has also been suggested as a bioindicator of groundwater quality [17–19]. Identifying suitable biomonitors of groundwater-dependent ecosystems including the hyporheic zone may become a priority for regulatory agencies, in particular due to legal drivers including the EU Water Framework Directive [20–22].

Despite increasing interest, sampling the invertebrate communities within the hyporheic zone remains challenging, since subsurface sediments are inherently difficult to access [23]. Freeze-coring [24], standpipe corers [25] and colonization devices [26] provide quantitative samples, but all have recognized limitations: freeze-coring is expensive, labour-intensive and causes extensive disturbance to the streambed, precluding repeated sampling from one location; standpipe corers collect a small sample (25 cm^3^), which may not effectively characterize the community present; and colonization devices require sediment excavation and installation followed by a colonization period that precedes sample collection [27–28].

Of the semi-quantitative methods available, two pump-sampling techniques are commonly used to sample invertebrates from hyporheic sediments: Bou-Rouch (BR) [29] and vacuum-pump (VP) sampling [30]. Pump-sampling techniques have several advantages over other methods: disturbance to the sediments is limited and repeated sampling from the same points is therefore possible; no recovery period is required, and both rapid surveys and long-term studies can therefore be conducted; both spatial and temporal variability in community composition can be characterized [31, 32]; sample collection is rapid (seconds to minutes); and the price of equipment is modest, with minimal ongoing sampling costs. Equally, pump-sampling techniques share limitations, including a bias towards the collection of smaller, less tenacious invertebrates [27, 33]; collection of only a proportion of the taxa and individuals present [12]; and the unknown size and position of the sampled sediments [34]. However, although BR suction forces are often informally described as greater than those of the VP, no study has compared assemblages sampled by these two techniques. The most effective pump-sampling technique to characterize hyporheic communities is therefore unknown.

We compared the BR and VP sampling techniques, examining their characterization of the macroinvertebrate component of the hyporheic invertebrate community, and their estimation of taxa richness and abundance. We focused on macroinvertebrates (rather than the full hyporheic assemblage including meiofauna), because benthic macroinvertebrate taxa are established as statutory Water Framework Directive biomonitors in EU surface waters [35, 36]. In contrast, research into the use of meiofauna as biomonitors is in its infancy [37] and such biomonitoring is not yet required under EU law, despite the functional importance of meiofauna in hyporheic communities [38]. We used both pump-sampling techniques to collect replicate samples in three contrasting temperate-zone streams in each of two biogeographical regions. Our first hypothesis was that abundance and richness estimates would be higher in BR samples than in VP samples, with rare taxa (i.e. those occurring at low abundance) most likely to be absent from VP samples. Our second hypothesis was that the assemblages sampled by the two techniques would be comparable, and that both techniques would consistently identify spatial (between-stream) variability in overall assemblage composition. This hypothesis of comparable assemblage composition was based on the size bias associated with pump sampling being common to both techniques, and on taxa not sampled by either technique being rare.

## Materials and Methods

Comparable field sampling campaigns were conducted in each of two temperate-zone biogeographical regions: southeast France (Continental region) and central England, UK (Atlantic region) [39]. During the Würm / Devensian glacial maxima, greater coverage of the UK by ice resulted in long-term reductions in biodiversity, whereas tundra vegetation dominated in France and more taxa were able to persist; these regions therefore have overlapping but distinct faunas [39–41].

### Study sites

**Fig 1:**
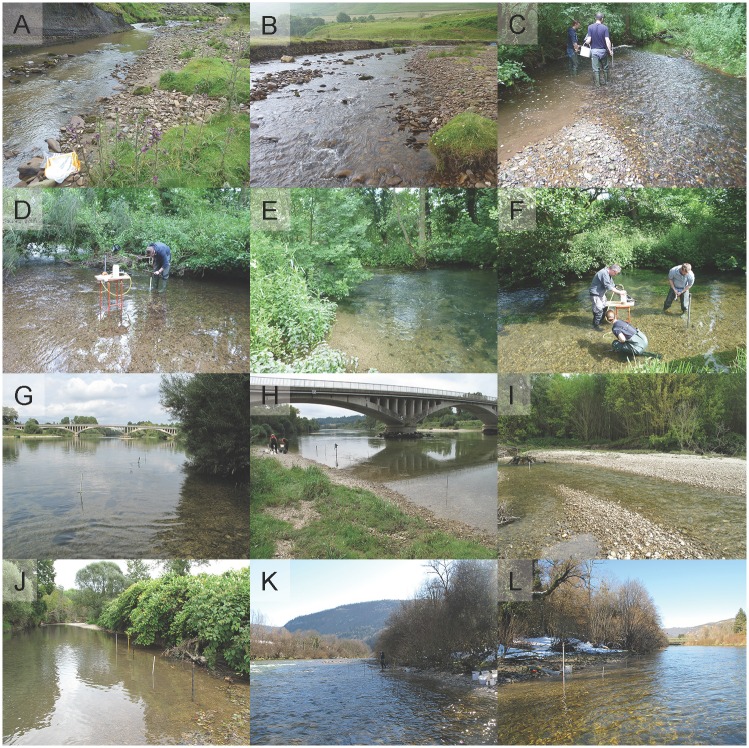
Hyporheic macroinvertebrate sampling sites on six streams in (A-F) the UK and (G-L) France. River Ashop (A) coarse-grained and (B) fine-grained sites; Black Brook (C) coarse and (D) fine sites; River Lathkill (E) coarse and (F) fine sites; Ain River (G) coarse and (H) fine sites; Albarine River (I) coarse and (J) fine sites; Bienne River (K) coarse and (L) fine sites.

In each region, sampling was conducted in three streams, selected for their contrasting physiographic characteristics, which allowed comparison of performance in varying environments and therefore facilitated inference of general patterns. Our previous experience working in these streams indicated that substrate composition was suitable for pump-sampling, and that hyporheic invertebrate communities were sufficiently abundant and diverse to allow comparison among systems. In the UK, the River Ashop, Black Brook and the River Lathkill were studied (Fig 1; Table 1). The Ashop is underlain by coarse-grained sandstone (Millstone Grit) and shale, and land use adjacent to the channel is low-intensity grazing, with scattered wetland vegetation (e.g. *Juncus*). Black Brook is underlain by Mercia Mudstone, with superficial sand and gravel deposits. This catchment is dominated by high-intensity cereal farming, with riparian woodland separating the stream from farmland on both banks. The Lathkill is underlain by karst limestone, surrounding land use is mainly low-intensity grazing, and riparian woodland lines the banks (Table 1).

**Table 1:** Characteristics of the UK (Ashop, Black Brook, Lathkill) and French (Ain, Albarine, Bienne) study streams, including hyporheic water quality and surface substrate characteristics at coarse-grained (C) and fine-grained (F) sites.

	Ashop	Black Brook	Lathkill	Ain	Albarine	Bienne
**Location**	Dark Peak area, Derbyshire	Loughborough, Leicestershire	White Peak area, Derbyshire	Gévrieux	Gévrieux, near Ain confluence	Vaux-lès-Saint-Claude, Franche Comté
**Latitude / longitude**	53.40 / -1.78	52.78 / -1.26	53.18 / -1.66	45.96 / 5.25	45.96 / 5.25	46.35 / 5.75
**Strahler order**	3	3	3	5	3	4
**Land use**	Rough pasture	Arable / urban	Pasture / woodland	Meadows / arable	Meadows / arable	Forest / pasture
**Geology**	Medium fluvial deposits (sandstone)	Fine fluvial deposits (mudstone)	Medium fluvial deposits (limestone)	Coarse fluvio-glacial deposits	Coarse fluvio-glacial deposits	Coarse alluvial fluvio-glacial deposits
**Catchment area (km**^**2**^**)**	45	44	64	3765	313	791
**Elevation (m)**	237	46	115	220	220	337
**Mean annual discharge (m**^**3**^ **s**^**-1**^**)**	1.4	0.4	1.3	123	7	30
**Channel width (m)**	6	3	5	70	15	38
**WATER QUALITY**^1^						
**Temperature (°C)**	12.8 ± 0.09	17.1 ± 0.03	13.7 ± 0.27	14.9 ± 0.39	12.8 ± 0.06	9.9 ± 0.17
**Conductivity (μS cm**^**-1**^**)**	64 ± 6	876 ± 9	551 ± 6	477 ± 28	409 ± 1	387 ± 4
**pH**	7.6 ± 0.05	7.5 ± 0.07	7.6 ± 0.07	NA	7.8 ± 0.05	7.5 ± 0.05
**Dissolved oxygen (mg L**^**-1**^**)**	12.1 ± 0.36	5.8 ± 0.73	9.6 ± 0.20	3.3 ± 0.23	9.1 ± 0.35	7.1 ± 0.07
**SUBSTRATE CHARCTERISTICS**^2^						
**Mean grain size (cm)**^**1**^	C: 2.7 ± 1.7; F: 2.1 ± 1.4	C: 3.7 ± 1.9; F: 1.6 ± 0.85	C: 2.4 ± 1.4; F: 2.1 ± 1.4	C: 5.3 ± 0.04; F: 4.3 ± 0.04	C: 4.9 ± 0.04; F: 4.4 ± 0.03	C: 6.1 ± 0.03; F: 5.1 ± 0.04
**D**_**50**_ **(cm)**	C: 1.6 / F: 1.6	C: 2.2 / F: 1.6	C: 3.2 / F: 1.6	C: 5.5 / F: 4.5	C: 5.0 / F: 4.5	C: 6.0 / F: 5.0
**D**_**84**_ **(cm)**	C: 4.5 / F: 3.2	C: 6.4 / F: 2.2	C: 4.5 / F: 3.2	C: 6.0 / F: 5.0	C: 5.5 / F: 5.0	C: 6.5 / F: 6.0

^1^Mean ± 1 SE, *n* = 8 for water quality measurements;

^2^Based on a modified Wolman pebble count of 100 grains

In France, the Ain River and two of its tributaries, the Bienne and Albarine Rivers were sampled (Fig 1; Table 1). These streams, located in the Meridional Jura region, are karstic over much of their course and differ considerably in size and discharge (Table 1). The lower Ain and lower Albarine meander across a wide alluvial valley filled by deep accumulations of coarse-grained Würm deposits; study sites were located in this area, close to the streams’ confluence. The Ain-Bienne confluence is >70 km upstream of the Ain-Albarine confluence. The Bienne flows through a narrow valley filled by very coarse fluvio-glacial deposits. Catchment land use is dominated by forest and pasture in all streams, although intensive arable farming is common in the lower Ain catchment (Table 1).

### Field sampling

Samples were collected between late June and mid-July (i.e. summer) 2014 in the UK, in early September 2014 in the Ain and Albarine, and in mid-February 2015 in the Bienne. High flows prevented concurrent sampling of all streams, but the variable timing did not compromise our sampling design because BR and VP samples were collected concurrently in each individual stream. Two sites within 500 m were selected on each stream, to represent the range of substrate characteristics present (i.e. one ‘coarse-grained’ and one ‘fine-grained’ site; Fig 1; Table 1). Landowners granted permission to access Ashop and Lathkill sites on private land, whereas all Ain, Albarine, Bienne and Black Brook sites were on public land. No permits were required to access any site, no land was protected, and no protected species were sampled. In total, 96 macroinvertebrate samples were collected: 2 regions × 3 streams × 2 sites × 2 techniques × 4 replicate samples per technique.

Working from downstream to upstream to minimize pre-sampling disturbance, four samples were collected using each technique at each site. The eight sampling points were positioned at longitudinal intervals of ~2 m, to avoid sampling activity disturbing subsequently sampled sediments. The technique used was alternated at successive sampling points, to prevent longitudinal changes in community composition being interpreted as an effect of the technique. Sampling points were preferentially selected in riffle habitats and the thalweg avoided, for consistency and to maximize richness estimates. Samples were collected immediately after insertion of the BR standpipe or VP sampling well, as immediate sample collection is an intrinsic feature of rapid survey techniques; in addition, Hunt and Stanley [42] found no difference in abundance or richness between BR samples from pre-installed standpipes and from those inserted immediately before sample collection.

**Fig 2:**
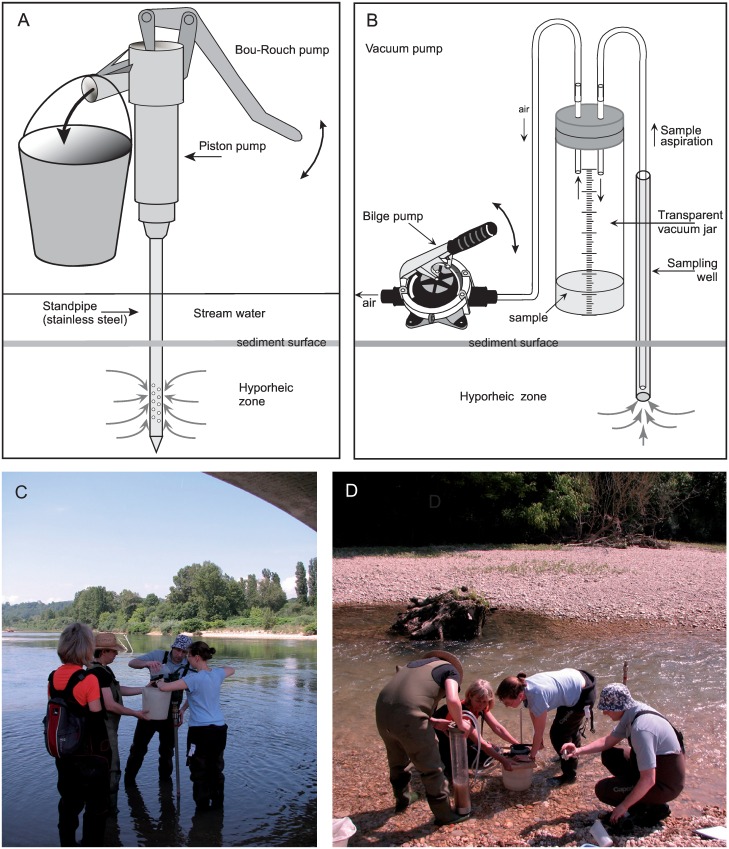
Equipment and mode of operation for two hyporheic macroinvertebrate pump-sampling techniques. (A) The Bou-Rouch pump (not to scale); (B) the vacuum pump (not to scale); (C) priming the Bou-Rouch sampling apparatus with filtered stream water in the Albarine River; and (D) vacuum-pump sample collection in the Albarine.

The BR sampling equipment included a 125-cm long steel standpipe with a 2-cm internal diameter and a 15-cm section perforated by 5-mm diameter holes. At each sampling point, this standpipe was inserted into the bed until the top of the perforated section was 15 cm below the substrate surface. A 0.5-L volume of stream water was filtered through a 200-μm net then used to prime the apparatus. A piston pump was then manually operated at the fastest rate possible, to extract 6.5 L of water (6 L hyporheic water and 0.5 L priming water), this volume being selected as typical [32, 43–45] (Fig 2A). Sediments in a collected sample were disturbed vigorously then all water poured through the 200-μm net into a second container, to retain invertebrates within the net. This process was repeated as required to retain all sampled invertebrates.

VP sampling wells each comprised a PVC pipe (19 mm internal diameter) which was placed onto the end of a stainless steel T-bar for insertion into the bed to a depth of 22.5 cm (i.e. the mid-point of the BR sampling depth range; Fig 1D). A length of hose was inserted into the sampling well and a bilge pump operated manually at the fastest rate possible to extract three 2-L aliquots (i.e. one 6-L sample) from the well base (Fig 2B). Samples were preserved in ≥70% industrial methylated spirits.

Temperature (°C), pH, conductivity (μS cm^-1^) and dissolved oxygen (DO; mg L^-1^) were measured in hyporheic water at each sampling point. Measurements were taken using standard instrumentation (Hanna Instruments, Leighton Buzzard, UK). Surface sediments were characterized at each site using a modified version of the pebble count method of 100 grains [46], with the transect being the approximate length of the sampling area (i.e. ~14 m).

### Macroinvertebrate identification

All macroinvertebrates were removed from samples and identified to the lowest taxonomic resolution practicable. In some cases (estimated as <10% of specimens), damage caused by passage through the BR apparatus prevented precise identification, and identification of some insects was limited by the early instar.

### Data analysis

All analysis of UK and French assemblages was conducted separately, to allow comparison of patterns in two regions with overlapping but distinct faunas. In calculating richness, the finest taxonomic resolution achieved was used, even if this varied between regions. Where organisms in one sample were identified to multiple taxonomic resolutions (e.g. *Niphargus* sp. and *Niphargus rhenorhodanensis*), only the finest was included in richness calculations, to avoid overestimation. In UK samples, different life stages (e.g. larval and adult beetles) were counted as separate taxa, because their different characteristics (e.g. morphology and behaviour) may have affected their capture during sampling.

Preliminary one-way analysis of similarities (ANOSIM) tests, conducted separately for each stream with “site” as the factor, indicated differences in assemblage composition for the coarse and fine Albarine (R = 0.243, P = 0.002) and Bienne (R = 0.550, P = 0.001) sites (hereafter termed e.g. Albarine-coarse). The two Ain, Ashop, Black Brook and Lathkill sites were therefore pooled in subsequent analyses and between-stream differences considered, whereas between-site differences were examined for the Albarine and Bienne (unless otherwise stated).

Univariate analyses were conducted in IBM SPSS Statistics Version 22 (IBM Corporation, New York, USA), with ln(*x*+1)-transformed abundance data and arc-sine square-root transformed proportional data. Multivariate analyses were done in PRIMER version 6.1.15 (PRIMER-E, Ivybridge, UK) on 4√(x)-transformed abundance data. EstimateS version 9.1.0 [47] was used to produce rarefaction curves and to calculate non-parametric richness estimators (NPREs).

#### Hypothesis 1. Differences in abundance and richness between techniques

Total abundance, the abundance of common taxa and taxa richness were calculated for each sample. ‘Common’ taxa were defined as accounting for >1% of all specimens and occurring in ≥25% of samples collected across all streams in a region. To compare estimates obtained by the two techniques across streams/sites and to identify technique*stream/site interactions, two-way ANOVA tests with post-hoc Tukey’s tests were conducted, with ‘technique’ (BR, VP) and ‘stream/site’ (i.e. Ain, Albarine-coarse, Albarine-fine, Bienne-coarse, Bienne-fine *or* Ashop, Black Brook, Lathkill) as fixed factors, and with the measure of abundance or richness as the dependent variable. Where interactions were identified, separate one-way ANOVAs were done for each stream/site, with ‘technique’ as a fixed factor.

Rarefaction was used to compare richness estimates obtained by the two techniques [48] based on sub-samples of pooled taxa richness [47]. Rarefaction curves were generated based on sample-based incidence data to allow comparison of the techniques in relation to sampling efficiency (defined as the number of taxa collected in relation to sampling effort, i.e. per 6-L sample in this study) [49]. Macroinvertebrate abundance differed in BR and VP samples (see below) and because collection of more individuals will reveal more taxa, curves were also generated based on sample-based incidence data rescaled to a common x-axis of individual occurrences [50, 51]. Rarefaction was conducted at the stream-level (not the site-level) for all streams including the Albarine and Bienne, to ensure comparability of sampling effort. Curves with 95% confidence intervals (CIs) were generated separately for each technique and each stream, to allow comparison of technique-specific richness estimates independent of stream-specific differences. Where BR and VP CIs did not overlap, differences between techniques were considered significant [51].

The abundance-based NPREs Chao1 [47] and Abundance Coverage-based Estimator (ACE) [52], and the incidence-based estimators Chao2 [47], Jackknife1 [53], Jackknife2, Bootstrap [54] and Incidence Coverage-based Estimator (ICE) [52] were calculated with 100 randomizations for each stream, to compare estimates of asymptotic taxa richness between techniques. The upper abundance limit for rare taxa was set at 10 individuals for ACE and ICE, or lower where this default value was not met. The bias-corrected forms of Chao1 and Chao2 were used except where coefficients of variation exceeded 0.5, in which case estimates were recalculated using the “classic” formula and the larger of Chao1 and ACE / Chao2 and ICE considered, respectively [47]. A two-way repeated-measures ANOVA was done with NPRE values for the eight replicate samples as within-subjects variables, and with ‘technique’ and ‘NPRE’ as between-subjects factors. Mauchly’s test was used to test the assumption of sphericity and where this was not met, Greenhouse-Geisser-adjusted P-values were consulted. Where interactions were identified, separate one-way ANOVAs were conducted for each stream, with ‘technique’ as a fixed factor.

#### Hypothesis 2. Differences in assemblage composition within and between streams and techniques

To assess differences in the assemblage composition characterized by the two techniques in the three streams, samples were ordinated using non-metric multidimensional scaling (NMDS) based on a Bray-Curtis similarity matrix, with 100 random restarts. The two-dimensional solution with the lowest stress was retained. Samples containing no macroinvertebrates were excluded from this analysis (i.e. 3 Ashop VP, 2 Lathkill VP and 1 Lathkill BR sample).

To test whether each technique characterized the assemblage consistently, the Index of Multivariate Dispersion (IMD) [55] was calculated for each individual stream/site with ‘technique’ as a factor. The IMD measures β diversity, expressing variability in assemblage composition as the average dissimilarity between individual samples and their group centroid in an ordination space. One-way ANOVA was conducted with IMD values for each technique-stream/site group as the dependent variable and with ‘technique’ as the factor.

To determine whether each technique identified differences in assemblage composition between streams/sites, separate one-way ANOSIM analyses with pairwise tests were done for BR and VP samples with ‘stream/site’ as a factor. To test whether assemblages characterized by the two sampling techniques in the three streams were comparable, a two-way crossed ANOSIM test was performed with ‘technique’ and ‘stream/site’ as factors. The main taxa accounting for differences between techniques and between streams/sites were determined using one-way similarity percentages (SIMPER) analysis. Finally, to examine the contribution of common taxa to the assemblages sampled by each technique, the abundance of each common taxon was calculated as a proportion of total abundance, and these proportions used as dependent variables in ANOVA tests, as described above.

## Results

### Environmental characteristics

In the UK, hyporheic water was cool, low-conductivity and well-oxygenated in the Ashop, which contrasted with the warmer, high-conductivity, lower-DO water of Black Brook; intermediate values were recorded in the Lathkill (Table 1). Surface sediments were dominated by coarse to very coarse gravel (sensu Gordon et al.) [56] in all streams, with greater variability between the two Black Brook sites than between streams (Table 1).

In France, Ain hyporheic water differed from the two other streams due to relatively high conductivity and very low oxygenation. DO concentrations were highest in the Albarine, whereas water temperatures were coolest in the Bienne. Surface sediments were dominated by coarse to very coarse gravel in all streams (Table 1).

### Assemblage composition

**Fig 3:**
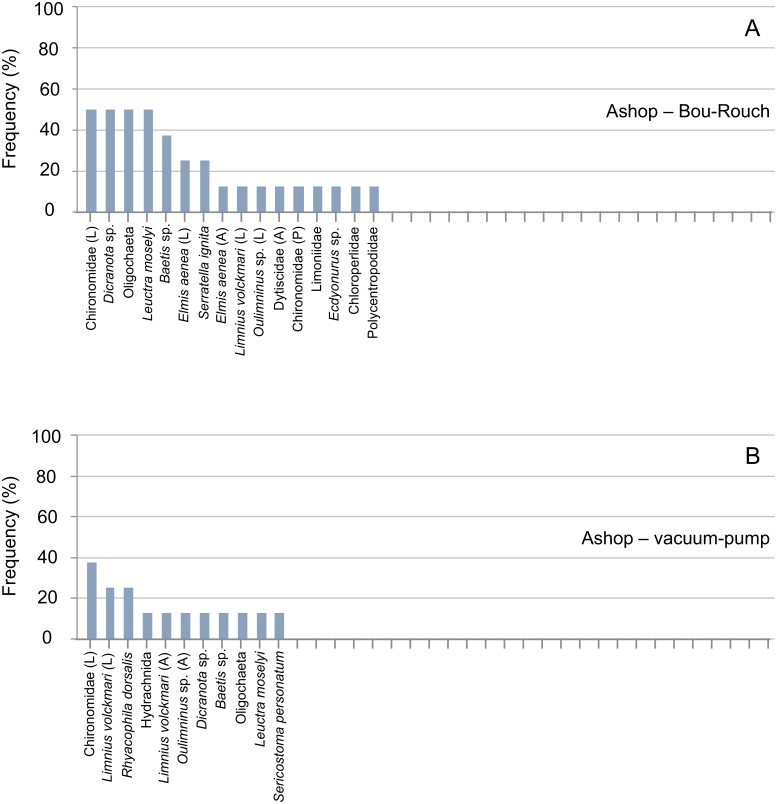
Rank frequency distribution (%) of macroinvertebrate taxa collected using Bou-Rouch (A) and vacuum-pump (B) hyporheic sampling techniques in the River Ashop, UK. A, adult; L, larvae; P, pupae.

**Fig 4:**
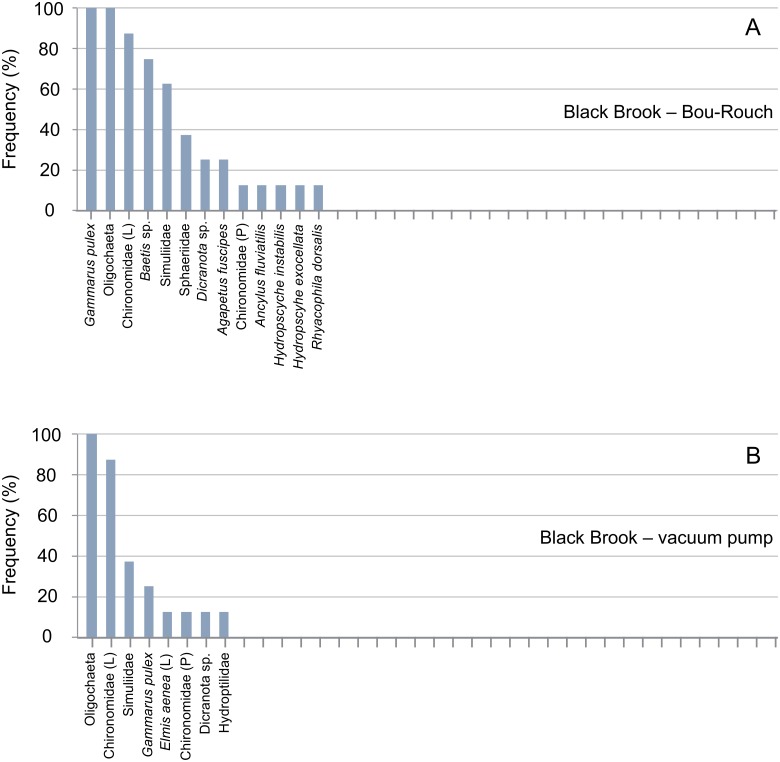
Rank frequency distribution (%) of macroinvertebrate taxa collected using Bou-Rouch (A) and vacuum-pump (B) hyporheic sampling techniques in the Black Brook, UK. A, adult; L, larvae; P, pupae.

**Fig 5:**
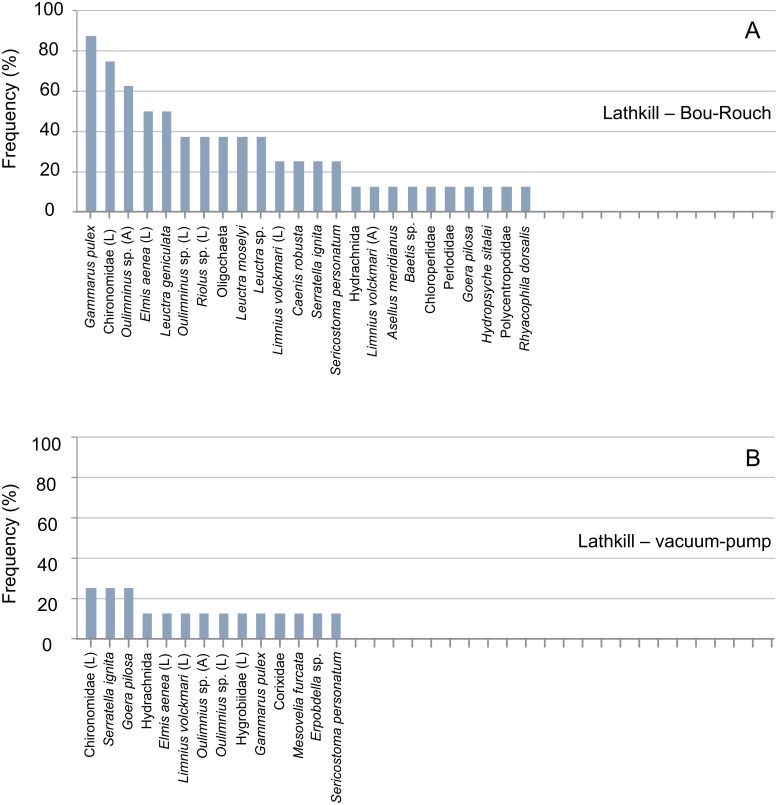
Rank frequency distribution (%) of macroinvertebrate taxa collected using Bou-Rouch (A) and vacuum-pump (B) hyporheic sampling techniques in the River Lathkill, UK. A, adult; L, larvae; P, pupae.

In total, 871 macroinvertebrates from 40 taxa were captured in 48 UK samples (S3 Table). Abundance was highest in Black Brook (mean ± SE, 35 ± 12 individuals [ind.] 6 L^-1^), and lower in the Lathkill (14 ± 4.4 ind. 6 L^-1^) and the Ashop (5.6 ± 1.5 ind. 6 L^-1^; two-way ANOVA, *F* = 13.12, df = 2, P < 0.001). Richness was comparable in all UK streams (3.0–4.8 ± 0.6–1.0 taxa 6 L^-1^; two-way ANOVA, *F* = 2.61, df = 2, P = 0.085). Total richness was highest in the Lathkill (28 taxa) and lowest in Black Brook (15 taxa). Oligochaeta and Chironomidae dominated the UK assemblage, comprising 43% and 21% of all macroinvertebrates, respectively (S1 Table); these taxa and *Gammarus pulex* (5.6%) were classified as common and were also among the most frequently collected taxa (Figs 3–5). *Leuctra* spp. (including *L*. *geniculata*, *L*. *moselyi* and early instars identified to genus) and *Baetis* sp. were also sampled regularly, each accounting for >2% of macroinvertebrates. Dominant taxa varied between streams: *Leuctra* spp., Chironomidae and *G*. *pulex* dominated in the Lathkill, the former accounting for 35% of the assemblage in this stream. Chironomidae, *Dicranota* sp. and *Leuctra* spp. dominated the Ashop assemblage, and Oligochaeta accounted for 63% of Black Brook macroinvertebrates (Figs 3–5).

**Fig 6:**
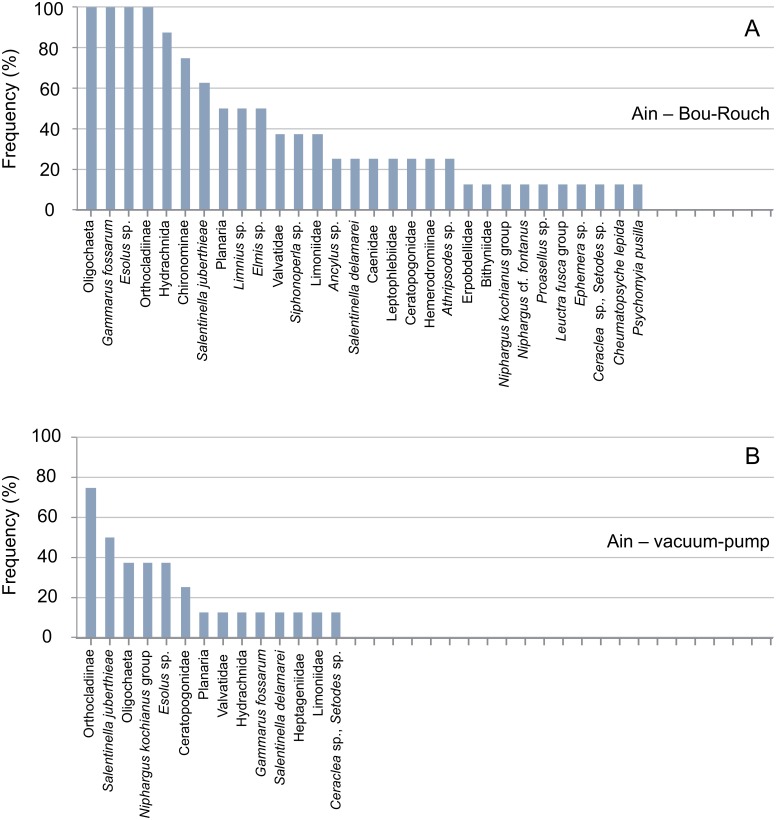
Rank frequency distribution (%) of macroinvertebrate taxa collected using Bou-Rouch (A) and vacuum-pump (B) hyporheic sampling techniques in the Ain River, France. A, adult; L, larvae; P, pupae.

**Fig 7:**
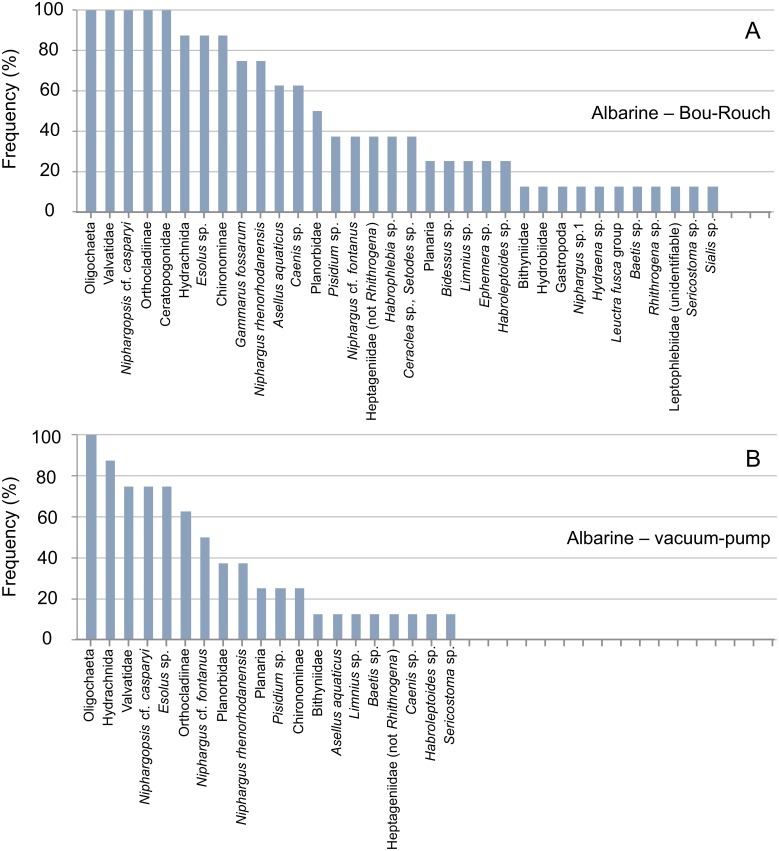
Rank frequency distribution (%) of macroinvertebrate taxa collected using Bou-Rouch (A) and vacuum-pump (B) hyporheic sampling techniques in the Albarine River, France. A, adult; L, larvae; P, pupae.

**Fig 8:**
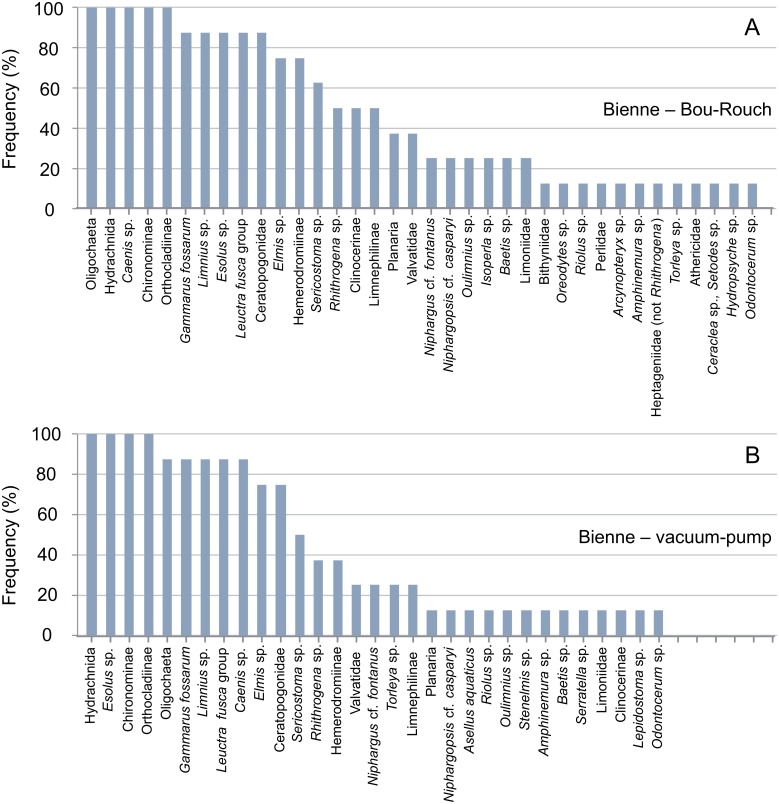
Rank frequency distribution (%) of macroinvertebrate taxa collected using Bou-Rouch (A) and vacuum-pump (B) hyporheic sampling techniques in the Bienne River, France. A, adult; L, larvae; P, pupae.

In total, 11,504 macroinvertebrates from 63 taxa were collected in 48 French samples (S4 Table). Abundance was lowest at Bienne-coarse (104 ± 21 ind. 6 L^-1^) and in the Ain (138 ± 58 ind. 6 L^-1^), and was higher at Albarine-fine (465 ± 156 ind. 6 L^-1^) and Bienne-fine (315 ± 45 ind. 6 L^-1^; two-way ANOVA post-hoc Tukey’s tests, P < 0.001). In total, 40, 34 and 30 taxa occurred in the Bienne, Albarine and Ain, respectively, and mean richness was lower in the Ain and at Albarine-coarse (7.9 ± 1.3–1.4 taxa 6 L^-1^) compared to Albarine-fine and both Bienne sites (13.8–16.5 ± 1.1–1.6 taxa 6 L^-1^; two-way ANOVA post-hoc Tukey’s tests, P ≤ 0.001). Orthocladiinae and Oligochaeta dominated, accounting for 26% and 25% of the assemblage, respectively, and being among the most frequently sampled taxa (Figs 6–8). *Niphargopsis* cf. *casparyi* (11%), *Esolus* sp. (10%), Valvatidae (6.1%), Hydrachnida (5.3%), Chironominae (2.6%), *Gammarus fossarum* (2.2%), *Leuctra fusca* group (1.8%) and *Limnius* sp. (1.1%) were also common (S1 Table). Dominant taxa differed between streams/sites: Orthocladiinae, Oligochaeta and *Esolus* sp. collectively comprised 85%, 69% and 63% of the Ain, Albarine-fine and Bienne-fine assemblages, respectively; *N*. cf. *casparyi*, Valvatidae and Oligochaeta accounted for 75% of the Albarine-coarse assemblage; and 61% of the Bienne-coarse assemblage was Orthocladiinae, Oligochaeta and Hydrachnida (Figs 6–8).

#### Hypothesis 1. Differences in abundance and richness between techniques

**Fig 9:**
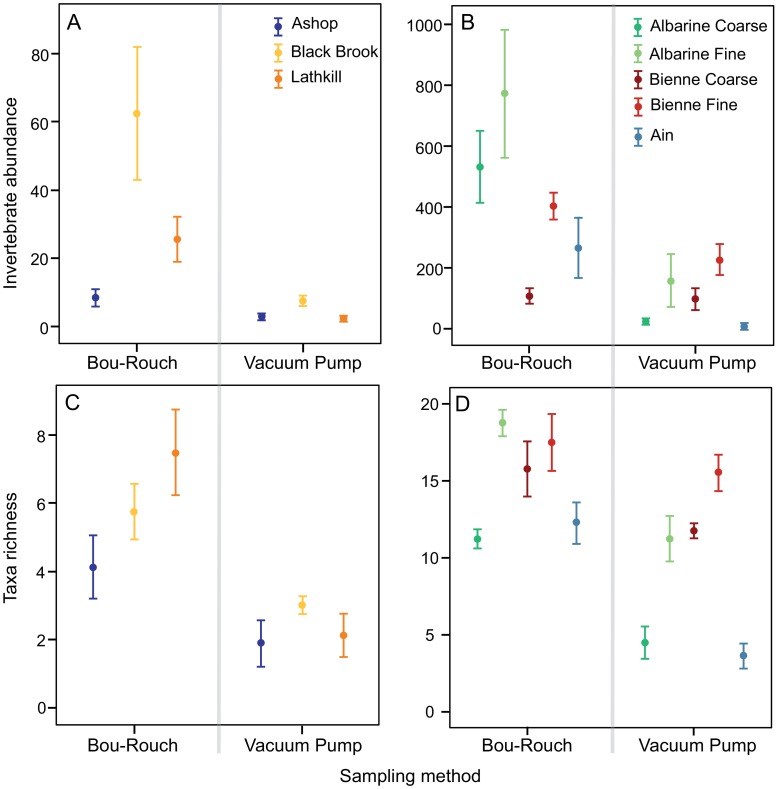
Hyporheic macroinvertebrate assemblage metrics estimated by Bou-Rouch and vacuum-pump sampling techniques. Mean ± 1 SE per 6-L sample: abundance in (A) UK and (B) French streams/sites; taxa richness in (C) UK and (D) French streams/sites. C, coarse-grained sites; F, fine-grained sites.

Total abundance was higher in UK BR samples than in VP samples and there was no technique*stream interaction (Fig 9A; S1 and S2 Tables). Abundance was also higher in French BR samples compared to VP samples, but the technique*stream interaction was significant (Fig 9B; S1 and S2 Tables): BR sampling collected more individuals than VP sampling in the Ain and at both Albarine sites, whereas abundance was comparable between techniques at Bienne sites (S1 and S2 Tables).

Considering common UK taxa, oligochaete abundance was higher in BR than in VP samples and the technique*stream interaction was significant: due to low abundance in the Lathkill and Ashop, abundance only differed between techniques in Black Brook (S1 and S2 Tables). Chironomidae and *G*. *pulex* abundance were higher in BR than in VP samples across all UK streams (S1 and S2 Tables). Although the taxon was not common across streams, 9.6 ± 3.0 *Leuctra* ind. 6 L^-1^ occurred in Lathkill BR samples (77 individuals across seven samples), whereas the genus was absent from VP samples (S1 and S2 Tables). In France, Oligochaeta and Chironominae were the only common taxa to be more abundant in BR samples across streams/sites, whereas *L*. *fusca* and *Limnius* sp. abundances were comparable between techniques across sites (S1 and S2 Tables). A technique*site interaction was observed for *Esolus* sp., *G*. *fossarum*, Hydrachnida, *N*. cf. *casparyi*, Orthocladiinae and Valvatidae, because abundance was only higher in BR samples at some sites (S1 and S2 Tables). Notably, in the Ain, 73 *G*. *fossarum* (9.1 ± 3.7 ind. 6 L^-1^) were captured across all BR samples, whereas only one individual occurred in one VP sample.

In total, 58, 41, 35 and 23 taxa occurred in French BR, French VP, UK BR and UK VP samples, respectively (S3 and S4 Tables). Richness was higher in BR samples than in VP samples across all UK and French streams/sites (Fig 9C and 9D; S1 and S2 Tables). Accordingly, the incidence of taxa was generally higher in BR than in VP samples, except in the Bienne, where rank frequency distributions were more similar between techniques (Figs 3–8). In UK streams, seven taxa which were rare in BR samples (2–4 ind. in total) were absent from VP samples: *Agapetus fuscipes*, *Ancylus fluviatilis*, *Caenis robusta*, Chloroperlidae, Polycentropodidae, *Riolus* sp. larvae and Sphaeriidae (Figs 3–5). Similarly, nine rare taxa (2–13 ind.) occurred only in French BR samples: *Ancylus* sp., *Arcynopteryx* sp., *Athripsodes* sp., *Bidessus* sp., *Ephemera* sp., Hydrobiidae, *Isoperla* sp., Leptophlebiidae and *Siphonoperla* sp. (Figs 6–8). Single individuals of a further nine UK and 10 French taxa occurred in BR but not VP samples. No taxon was repeatedly sampled exclusively by the VP, although UK and French VP samples included single specimens from five and three taxa, respectively (S3 and S4 Tables).

**Fig 10:**
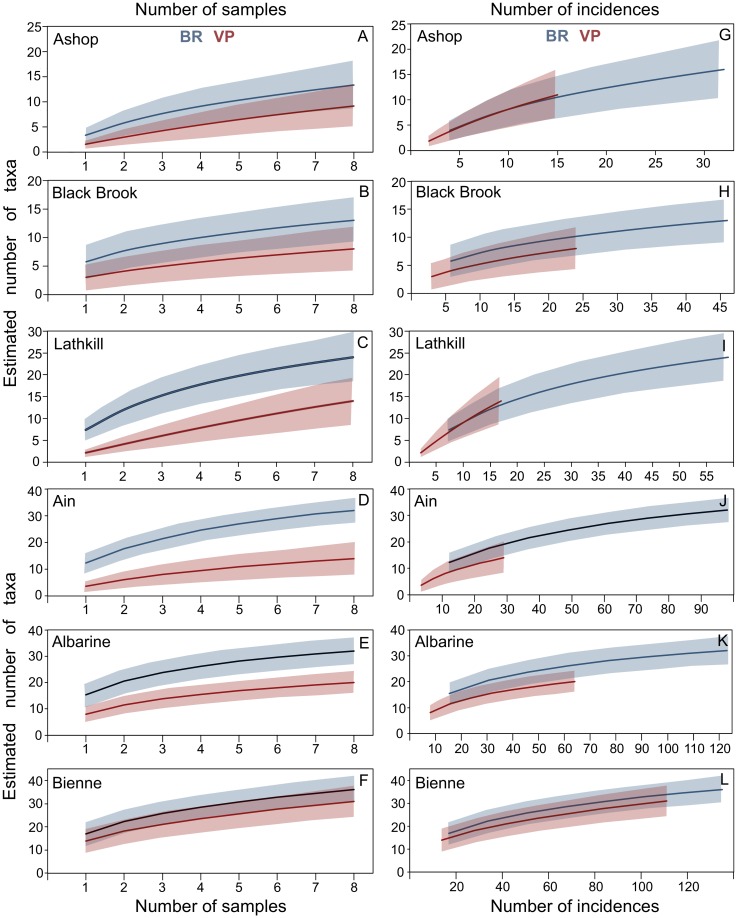
Rarefaction curves of taxa richness, for samples collected using two hyporheic macroinvertebrate pump-sampling techniques, Bou-Rouch (BR, blue) and vacuum-pump (VP, red) sampling. Sample-based incidence curves for: (A) the Ashop; (B) Black Brook; (C) the Lathkill; (D) the Ain; (E) the Albarine; (F) the Bienne; and sample-based incidence curves rescaled to the number of incidences, for: (G) the Ashop; (H) Black Brook; (I) the Lathkill; (J) the Ain; (K) the Albarine; (L) the Bienne. To allow rescaling to produce smooth curves, the expected number of incidences in *t* samples was calculated as (*t* / total number of samples)*total number of incidences. Solid lines indicate the estimated number of taxa; shaded areas indicate upper and lower 95% CIs.

Rarefaction curves based on sample-based incidence data provided significantly higher richness estimates for BR than VP samples in the Lathkill (Fig 10C), Ain (Fig 10D) and Albarine (Fig 10E), whereas overlap of the lower BR and upper VP CIs indicated comparable estimates in the Ashop (Fig 10A), Black Brook (Fig 10B) and Bienne (Fig 10F). When curves were rescaled to account for differences between techniques in the number of incidences, richness estimates were comparable for the two techniques (Fig 10).

Based on seven NPREs, maximum estimates of asymptotic richness were higher for BR samples in the Ashop (55 taxa, Chao1), Albarine (44, Chao1) and Black Brook (20, Jackknife2), and were higher for VP samples in the Ain (46, Chao1), Bienne (73, Chao1) and Lathkill (40, ICE; Table 2). Estimates were comparable between techniques in both UK streams (two-way repeated-measures ANOVA, NPRE*technique, F = 2.74, df = 1.71, P = 0.082) and French streams (*F* = 2.80, df = 1.60, P = 0.082), and there were no samples*technique*NPRE interactions (UK: *F* = 1.37, df = 10.28, P = 0.223; France: *F* = 1.36, df = 9.63, P = 0.234), indicating that estimates were comparable according to all NPREs in both regions.

**Table 2:** Non-parametric estimators of macroinvertebrate taxa richness in hyporheic samples collected from three streams in the UK and France using Bou-Rouch and vacuum-pump sampling techniques.

	UK						France					
	Ashop		Black Brook		Lathkill		Ain		Albarine		Bienne	
Estimator	BR	VP	BR	VP	BR	VP	BR	VP	BR	VP	BR	VP
Chao1	***55***	16	12	*16*	32	26	36	***46***	44	*23*	41	***73***
Chao2	34	25	*18*	***15***	35	32	*39*	***42***	***41***	*25*	*47*	***49***
Jackknife1	24	18	17	12	33	24	42	21	40	26	47	42
Jackknife2	29	23	20	14	38	30	45	26	44	29	52	50
Bootstrap	19	14	15	10	28	18	37	17	36	23	41	36
ACE	*40*	19	15	*16*	30	32	37	*31*	39	***25***	46	*69*
ICE	28	29	*18*	*14*	35	40	***44***	*26*	*39*	***27***	*47*	*46*

BR, Bou-Rouch; VP, vacuum pump. Estimators were each calculated based on eight replicate samples. Chao1 and ACE were calculated using abundance data and other estimators using incidence data. Values in italics indicate estimates calculated using the classic formula; only the larger of Chao1 / ACE and of Chao2 / ICE (in bold) should be considered [47].

#### Hypothesis 2. Differences in assemblage composition within and between streams and techniques

**Fig 11:**
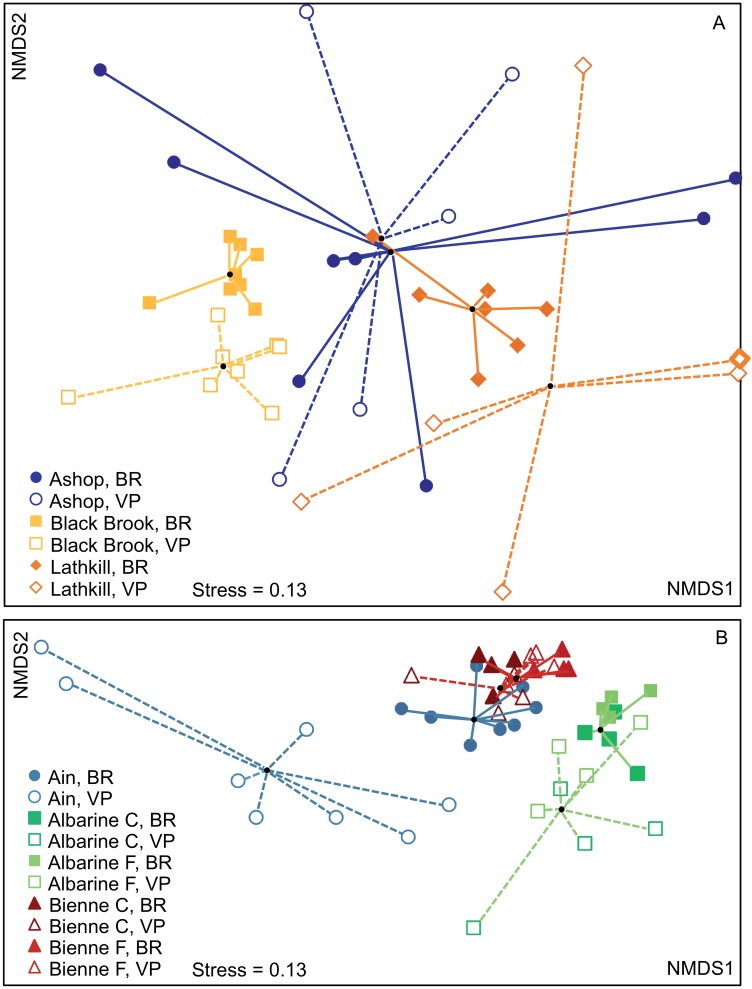
Non-metric multidimensional scaling ordination of macroinvertebrate assemblage composition characterized by two hyporheic pump-sampling techniques in three streams in: (A) the UK; (B) France. The two techniques are Bou-Rouch (BR) and vacuum-pump (VP) sampling. Albarine and Bienne samples are separated into coarse-grained (C) and fine-grained (F) sites due to differences in assemblage composition. Lines link individual samples to the stream/site-technique group centroid. *N* = 8 for Ain, Black Brook and Ashop BR; *n* = 7 for Lathkill BR; *n* = 6 for Lathkill VP; and *n* = 5 for Ashop VP stream-technique groups (other samples contained no macroinvertebrates); *n* = 4 for Albarine and Bienne site-technique groups. The high NMDS1 score of a Lathkill VP sample (indicated by a thick border) was reduced from 3.1 to aid presentation.

NMDS ordinations indicated differences in assemblage composition within and between techniques and streams/sites (Fig 11). On the UK ordination, Black Brook BR and VP samples plotted as relatively distinct, adjacent groups with low NMDS1 scores; Lathkill and Ashop samples were more widely dispersed, especially VP samples (Fig 11A). On the France ordination, Bienne samples formed a tight, overlapping group with high NMDS2 scores. BR samples from both Albarine sites also plotted as a distinct group adjacent to a more dispersed VP group, all with high NMDS1 scores. Ain VP samples were dispersed along NMDS1, whereas Ain BR samples overlapped with the Bienne cluster (Fig 11B). On both ordinations, greater dispersion was associated with lower abundance, for example mean ± SE abundance was 8.5 ± 2.4 and 2.2 ± 0.7 individuals per sample for the dispersed Ain and Lathkill VP sample groups, respectively, compared to 26 ± 6.7 and 267 ± 99 individuals per sample for the tighter BR groups in these rivers (S1 Table). The IMD was higher for VP than BR samples in the UK (1.2 ± 0.07 compared to 0.8 ± 0.07; one-way ANOVA, *F* = 20.05, df = 1, P = 0.011) and in France (1.2 ± 0.07 compared to 0.79 ± 0.07; *F* = 19.87, df = 1, P = 0.002).

BR assemblages differed among all UK streams (one-way ANOSIM Global R = 0.514) and all French streams/sites (R = 0.659), as did French VP assemblages (R = 0.398; pairwise tests, P ≤ 0.029). UK VP assemblages also differed among streams overall (Global R = 0.184, P = 0.001), but low-abundance Lathkill and Ashop assemblages were comparable (pairwise test, P >0.05). Assemblage composition differed in BR and VP samples in the UK (two-way crossed ANOSIM, Global R = 0.493, P = 0.001) and France (R = 0.481, P = 0.001). Higher abundance of Oligochaeta, Chironomidae, *G*. *pulex*, *Leuctra* spp. and *Baetis* sp. in BR samples collectively explained 50% of the UK difference, and higher abundance of Orthocladiinae, *Esolus* sp., Oligochaeta, *G*. *fossarum*, Hydrachnida, Chironominae and Valvatidae in BR samples accounted for 51% of the difference in France (SIMPER).

Considering the contribution of individual taxa to the UK assemblage, only oligochaetes accounted for a higher proportion of the assemblage in BR compared to VP samples, and this pattern was only observed in Black Brook (63 ± 5.9% compared to 34 ± 4.6%; one-way ANOVA, *F* = 14.43, df = 1, P = 0.002). In France, the contribution to the assemblage was higher in BR compared to VP samples for Orthocladiinae (29 ± 4.2% compared to 21 ± 4.8%; two-way ANOVA, *F* = 4.30, df = 1, P = 0.045) and Chironominae (4.2 ± 1.0% compared to 2.0 ± 0.6%; *F* = 6.89, df = 1, P = 0.012).

## Discussion

Our equivalent sampling campaigns in France and the UK, in the Continental and Atlantic biogeographical regions respectively, revealed macroinvertebrate assemblages that shared major taxa (i.e. Oligochaeta, Chironomidae, *Gammarus* and *Leuctra*) but were otherwise different. Higher abundance and taxa richness characterized French assemblages, and they featured a rich stygobite fauna, which was not recorded in the UK (S3 and S4 Tables). These faunal contrasts may be partly explained by Europe’s glacial history and the associated increase in richness with progression from northwest to southeast [57, 58]. In addition, spacious hyporheic interstices in the coarse fluvio-glacial sediments of French streams [59, 60] would have facilitated faster sample extraction using any pump-sampling technique, allowing the capture of more organisms [42]. Despite these differences, patterns of assemblage characterization in BR and VP samples were largely comparable across regions.

### Hypothesis 1. Differences in abundance and richness between techniques

More taxa were sampled at higher abundances using the BR compared to the VP technique, supporting our first hypothesis. Despite variation in assemblage composition within and between streams and regions, higher richness and abundance characterized most BR samples, and abundance was higher in BR samples for all UK and some French common taxa. We attribute these differences to the faster pumping rate achieved by BR compared to VP sampling (0.43 L s^-1^ compared to 0.17 L s^-1^) and the greater suction forces therefore exerted by the BR pump [42]. Only at the low-abundance Bienne-coarse site was abundance comparable between techniques. The grain size of surface sediments at Bienne-coarse was the greatest of any site, and larger interstices may have increased VP pumping and capture rates, rendering the two techniques more comparable [42], as well as potentially reducing macroinvertebrate attachment to sediment grains to evade capture, facilitating movement of organisms through interstitial pathways, and altering faunal depth distributions.

Sixteen and 19 taxa that occurred at low abundance in UK and French BR samples, respectively, were absent from VP samples. Some frequently observed taxa were also absent from VP samples, for example, the genus *Leuctra* was not collected in Lathkill VP samples despite 77 individuals occurring in Lathkill BR samples. In contrast, numerous *L*. *fusca* group nymphs occurred in both BR and VP Bienne samples. This difference between regions may reflect interspecific differences in morphology and behaviour: later-instar nymphs including the morphologically more-robust *L*. *geniculata* occurred in the Lathkill and may have been better adapted to resist weaker VP suction forces [61]. Similarly, 73 *G*. *fossarum* were captured across all BR samples in the Ain, whereas one individual was present in VP samples. In contrast, *G*. *fossarum* abundance was high and comparable between techniques in the Bienne. These contrasting capture rates may reflect seasonal variability in amphipod activity levels, with reduced activity and therefore reduced ability to evade capture in the Bienne, which was sampled in winter [62].

Despite mean richness being higher in BR samples and despite the absence of many taxa from VP samples, NPRE asymptotic richness estimates and rescaled rarefied richness estimates were comparable between techniques. This comparability indicates that, at an equivalent sampling effort, the capture of more individuals by the BR pump was responsible for its higher richness estimates, not a greater ability to collect certain macroinvertebrate taxa, for example those able to resist suction forces by clinging to sediment grains or by swimming out of the sampled water. Similarly, Dole-Olivier et al. [12] found no support for their prediction that invertebrate capture by BR sampling would depend on traits such as body size and ecology (e.g. obligate groundwater vs. primarily benthic taxa).

### Hypothesis 2. Differences in assemblage composition within and between streams and techniques

Our second hypothesis stated that assemblage composition would be comparable in BR and VP samples taken in a stream. We reject this hypothesis, because the two-way crossed ANOSIM indicated that assemblage composition differed between techniques in both regions. However, SIMPER indicated that higher abundance of common taxa in BR samples caused assemblages to differ from VP samples, not the collection of a different taxonomic assemblage. Equally, the proportion of the assemblage accounted for by individual common taxa differed between techniques for few taxa: Chironominae and Orthocladiinae in France and Oligochaeta in the UK. These observations support our suggestion (in hypothesis 2) that both techniques can identify major differences in the range of taxa present. Dole-Olivier et al. [12] also found that the dominant taxa observed in BR samples collected within the confines of a quantitative benthic sampler effectively reflected the dominant taxa present.

ANOSIM indicated that Ashop VP and Lathkill VP assemblages were comparable. However, we do not attribute this result to similar assemblages occurring in these streams. Instead, we suggest that VP sampling did not always identify differences in assemblage composition due to low abundance and associated within-stream variability. Equally, we suggest that the dispersed VP clusters on NMDS ordinations (e.g. for the Ain and Lathkill VP sample groups) do not indicate truly heterogeneous assemblages, but reflect the presence of few individuals. In contrast, when more individuals were captured (e.g. in the Bienne), VP sampling characterized assemblages with greater consistency and distinguished between streams.

Similarly, previous studies have demonstrated spatial and temporal changes in hyporheic invertebrate assemblages using VP sampling. For example, Boulton et al. [63] identified spatial differences in assemblages according to land use; Wood et al. [44] and Stubbington et al. [32, 64] explored temporal variability in assemblage composition in relation to surface flow; and Datry et al. [65] demonstrated assemblage variability in relation to longitudinal changes in flow permanence. To characterize a hyporheic community effectively using the VP technique, sufficient replicates should be collected [66], with the number required being determined during a preliminary examination of invertebrate abundance. A large sample volume may also be desirable where capture of all taxa including infrequent and rare taxa (i.e. richness estimation) is prioritized, although the decrease in abundance estimates associated with increasing sample volume may conflict with some study aims [42, 67].

Higher abundance across taxa, higher richness, lower IMD values, more distinct ANOSIM groups, and tighter NMDS groups were typically observed for BR samples in both regions. The presence of a more abundant, richer and less variable assemblage in BR samples indicated that this technique represented the hyporheic community more effectively than VP sampling. Capture of fewer individuals was the principal limitation of VP sampling and, at an equivalent sampling effort, resulted in the collection of fewer taxa, reduced within-stream consistency and reduced between-stream distinction, in particular in low-abundance UK streams.

### Recommendations for pump sampling of hyporheic macroinvertebrates

BR sampling provided higher abundance and richness estimates at a comparable sampling effort, and this technique may therefore be the most effective means of characterizing hyporheic macroinvertebrate assemblages during rapid surveys, for example for biomonitoring purposes (Table 3). BR sampling also distinguished between sites and streams more consistently, and it is therefore recommended for surveys seeking to characterize spatial heterogeneity in community composition (Table 3). BR sampling captured more rare taxa, and missed few taxa present in VP samples; this technique is therefore recommended for taxonomic and biogeographic surveys and other studies requiring comprehensive taxa lists and maximum richness estimates, and for studies examining the stygobite component of hyporheic communities, which may include many sporadically distributed and low-abundance taxa [41, 68]. Collection of such stygobites may be particularly important where these taxa function as bioindicators of ecosystem health [18, 19]. In addition, as the abundance and richness of a hyporheic community declines, the absence of each additional taxon from a sampled assemblage represents an increasing proportion of community richness. Therefore, VP sampling is less suitable in low-abundance and low-richness streams, such as those in the Atlantic biogeographical region, and more locally, those with finer sediments [60].

**Table 3:** Recommendations for use of Bou-Rouch or vacuum-pump sampling techniques in different types of hyporheic macroinvertebrate community surveys.

Survey type / requirements	Bou-Rouch	Vacuum pump
Estimation of abundance and taxa richness	✓	
Rapid surveys to examine community composition	✓	
Characterization of spatial heterogeneity in community composition	✓	
Collection of rare taxa	✓	
Characterization of temporal variability in community composition		✓
Collection of undamaged organisms		✓
Measurement of water chemistry		✓

Despite its apparent advantages, BR sampling also has limitations. Selection of the most appropriate pump-sampling technique therefore depends on the aims of an investigation, and VP sampling may be more suitable for some study types (Table 3). Firstly, BR standpipes are considerably more expensive than VP wells, which may restrict the number that can be installed semi-permanently to allow repeated sample collection and examination of temporal changes in community composition. Being less valuable and less conspicuous, installed VP wells are also less likely to be vandalised or stolen. Secondly, soft-bodied organisms, delicate groups including some stygofauna, and larger taxa including amphipods and isopods may all be damaged as they pass through the BR apparatus. Therefore, where precise measurement of all collected organisms or species-level identification of taxonomically demanding groups is needed, VP sampling may be preferable. Finally, the BR pump requires priming with filtered surface water at the onset of sampling, which precludes accurate measurement of physiochemical parameters in invertebrate sampling water (Table 3), although water can be extracted from a BR standpipe using a peristaltic pump [69].

Pump-sampling techniques share the limitation that the sediments sampled are unknown: collection points are fixed, but water may enter them from any direction [34]. The two techniques are therefore unlikely to have sampled the same sediment volume or shape, this source of variability being consistent across samples. Although this limitation may not be problematic in rapid surveys, where quantitative information about macroinvertebrate distribution is required, methods may need to be adapted and the sampled sediment volume extrapolated from porosity measurements [12, 70]. For precise characterization of the sampled sediment volume, measurements of flow circulation patterns are also required [70]. A smaller sample volume may also facilitate study of macroinvertebrate distribution within hyporheic sediments, as it ensures that water originates from a better-defined zone [71].

## Conclusions

With increasing legislative impetus for comprehensive biomonitoring of freshwater ecosystems, hyporheic invertebrate sampling methods require comparison and evaluation. Our study provides evidence that BR sampling captures more macroinvertebrate individuals and taxa more consistently, rendering assemblages in replicate samples more representative of instream communities. Further research is required to determine if these patterns also represent the meiofaunal component of hyporheic assemblages. VP sampling nonetheless remains an important option for inexpensive and rapid sample collection, and we recommend that results of VP studies be interpreted in light of limitations highlighted here.

## Supporting Information

S1 Table Mean ± 1 SE macroinvertebrate abundance, taxa richness, and abundance of common taxa in 6-L samples collected from all streams and three individual streams in (a) the UK and (b) France using Bou-Rouch (BR) and vacuum-pump (VP) pump-sampling techniques. ‘Common’ taxa are defined in the text. Coarse-grained and fine-grained sites are distinguished for the Albarine and the Bienne due to differences in assemblage composition between sites. Taxa are listed in order of decreasing abundance. Further taxonomic information is given in S3 and S4 Tables.
(DOCX)

S2 Table Results of two-way ANOVAs to determine differences in macroinvertebrate abundance, taxa richness and the abundance of common taxa between Bou-Rouch (BR) and vacuum-pump (VP) sampling techniques used across streams/sites in (a) the UK and (b) France. One-way ANOVA tests were conducted where technique*stream/site interactions were significant; df = 1. ‘Common’ taxa are defined in the text. Coarse-grained and fine-grained sites are distinguished for the Albarine and the Bienne due to differences in assemblage composition between sites. Taxa are listed in order of decreasing abundance. Further taxonomic information is given in S3 and S4 Tables.
(DOCX)

S3 Table The abundance of macroinvertebrate taxa in individual Bou-Rouch and vacuum-pump samples collected at coarse-grained and fine-grained sites on the River Ashop, Black Brook and River Lathkill (UK). (XLSX)

S4 Table The abundance of macroinvertebrate taxa in individual Bou-Rouch and vacuum-pump samples collected at coarse-grained and fine-grained sites on the Ain River, Albarine River and Bienne River (France). (XLSX)
