# Supplementary material for: Characterization of Macroinvertebrate Communities in the Hyporheic Zone of River Ecosystems Reflects the Pump-Sampling Technique Used
Source: PLoS One. 2016 Oct 10;11(10):e0164372. doi: 10.1371/journal.pone.0164372 (PMC5056715; doi:10.1371/journal.pone.0164372)
Supplement: S1 Table — ‘Common’ taxa are defined in the text. Coarse-grained and fine-grained sites are distinguished for the Albarine and the Bienne due to differences in assemblage composition between sites. Taxa are listed in order of decreasing abundance. Further taxonomic information is given in S3 and S4 Tables. (DOCX) [file pone.0164372.s001.docx]

**S1 Table. Mean ± 1 SE 6 L^-1^ macroinvertebrate abundance, taxa richness, and abundance of common taxa in samples** **collected from all streams and three individual streams in (a) the UK and (b) France using each of two pump-sampling methods.** BR, Bou-Rouch; VP, vacuum pump sampling methods. ‘Common’ taxa are defined in the text. Coarse-grained and fine-grained sites are distinguished for the Albarine and the Bienne due to differences in assemblage composition between sites. Taxa are listed in order of decreasing abundance. Further taxonomic information is given in Tables S3-S4.

|  | | **BR** | **VP** | | **BR** | **VP** | **BR** | **VP** | **BR** | **VP** | | **BR** | **VP** | **BR** | **VP** |
| --- | --- | --- | --- | --- | --- | --- | --- | --- | --- | --- | --- | --- | --- | --- | --- |
| **(a) UK** | **Total^1^** | **Ashop** | | | **Black Brook** | |  |  | **Lathkill** | | |  |  | **All streams** | |
| **Total abundance** | 871 | 8.4 ± 2.5 | | 2.8 ± 0.7 | 63 ± 19 | 7.5 ± 1.5 |  |  | 26 ± 6.7 | | 2.2 ± 0.7 |  |  | 32 ± 8.1 | 4.2 ± 0.8 |
| **Taxa richness** | 40 | 4.1 ± 0.9 | | 1.8 ± 0.7 | 5.8 ± 0.8 | 3.0 ± 0.3 |  |  | 7.5 ± 1.3 | | 2.1 ± 0.6 |  |  | 5.8 ± 0.6 | 2.3 ± 0.3 |
| **Oligochaeta** | 373 | 1.0 ± 0.5 | | 0.1 ± 0.1 | 42 ± 16 | 2.4 ± 0.6 |  |  | 1.5 ± 0.9 | | 0 |  |  | 15 ± 6.4 | 0.8 ± 0.3 |
| **Chironomidae** | 185 | 2.0 ± 1.0 | | 0.6 ± 0.3 | 13 ± 3.3 | 3.9 ± 1.0 |  |  | 3.5 ± 1.5 | | 0.3 ± 0.2 |  |  | 6.1 ± 1.6 | 1.6 ± 0.5 |
| ***Leuctra* spp.^2^** | 90 | 1.1 ± 0.5 | | 0.5 ± 0.5 | 0 | 0 |  |  | 9.6 ± 3.0 | | 0 |  |  | 3.6 ± 1.3 | 0.2 ± 0.2 |
| ***G. pulex*** | 49 | 0 | | 0 | 2.5 ± 0.8 | 0.3 ± 0.2 |  |  | 3.1 ± 1.3 | | 0.3 ± 0.3 |  |  | 1.9 ± 0.6 | 0.2 ± 0.1 |
| **(b) France** |  | **Ain** | | | **Albarine-coarse** | | **Albarine-fine** | | **Bienne-coarse** | | | **Bienne-fine** | | **All streams** | |
| **Total abundance** | 11504 | 267 ± 99 | | 8.5 ± 2.4 | 532 ± 119 | 26 ± 11 | 772 ± 210 | 159 ± 87 | 109 ± 24 | | 98 ± 37 | 403 ± 45 | 227 ± 50 | 391 ± 66 | 88 ± 23 |
| **Taxa richness** | 63 | 12 ± 1.3 | | 3.6 ± 0.8 | 11 ± 0.6 | 4.5 ± 1.0 | 19 ± 0.9 | 11 ± 1.5 | 16 ± 1.8 | | 12 ± 0.5 | 18 ± 1.8 | 16 ± 1.2 | 15 ± 0.8 | 8.4 ± 1.1 |
| **Orthocladiinae** | 3021 | 175 ± 87 | | 5.3 ± 2.9 | 32 ± 10 | 0.5 ± 0.5 | 189 ± 99 | 10 ± 5 | 43 ± 13 | | 21 ± 7.4 | 67 ± 15 | 34 ± 9 | 114 ± 35 | 12 ± 3.1 |
| **Oligochaeta** | 2873 | 22 ± 8.0 | | 27 ± 16 | 100 ± 30 | 8.8 ± 4.5 | 258 ± 130 | 46 ± 30 | 17 ± 4.7 | | 22 ± 8.2 | 133 ± 17 | 89 ± 20 | 92 ± 27 | 28 ± 8.5 |
| ***N. casparyi*** | 1298 | 0 | | 0 | 195 ± 68 | 0.8 ± 0.5 | 106 ± 62 | 22 ± 1.4 | 0 | | 0 | 0.5 ± 0.3 | 0.3 ± 0.3 | 50 ± 21 | 3.9 ± 1.7 |
| ***Esolus* sp.** | 1187 | 30 ± 8.9 | | 14 ± 9.0 | 9.3 ± 4.6 | 0.8 ± 0.5 | 112 ± 42 | 28 ± 16 | 6.8 ± 2.4 | | 5.8 ± 1.7 | 51 ± 7.2 | 23 ± 6.3 | 40 ± 10 | 9.6 ± 3.5 |
| **Valvatidae** | 701 | 0.5 ± 0.3 | | 18 ± 12 | 106 ± 42 | 10 ± 4.6 | 29 ± 11 | 27 ± 26 | 0 | | 0.5 ± 0.3 | 2.3 ± 1.3 | 0 | 23 ± 10 | 6.3 ± 4.3 |
| **Hydrachnida** | 604 | 12 ± 4.9 | | 7.3 ± 3.2 | 52 ± 24 | 3.5 ± 1.9 | 13 ± 8.2 | 11 ± 5.9 | 5.0 ± 2.0 | | 19 ± 9.6 | 17 ± 6.8 | 6.8 ± 2.3 | 19 ± 5.3 | 6.7 ± 2.2 |
| **Chironominae** | 301 | 5.3 ± 2.9 | | 0.6 ± 0.4 | 6.3 ± 6.0 | 0 | 14.5 ± 5.8 | 1.3 ± 0.8 | 7.8 ± 1.4 | | 5.3 ± 3.0 | 20 ± 4.9 | 10 ± 3.3 | 9.8 ± 2.0 | 2.8 ± 1.4 |
| ***G. fossarum*** | 252 | 9.1 ± 3.7 | | 0.1 ± 0.1 | 0.8 ± 0.5 | 0 | 4.8 ± 0.8 | 0 | 2.0 ± 0.7 | | 1.0 ± 0.4 | 19 ± 5.4 | 18 ± 3.5 | 7.4 ± 1.9 | 3.1 ± 1.4 |
| ***Leuctra fusca*** | 206 | 0.1 ± 0.1 | | 0 | 0.3 ± 0.3 | 0 | 0 | 0 | 7.5 ± 2.5 | | 11 ± 60. | 20 ± 10 | 13 ± 7.2 | 4.6 ± 2.1 | 4.0 ± 1.8 |
| ***Limnius* sp.** | 121 | 3.3 ± 2.3 | | 0.1 ± 0.1 | 0 | 0 | 0.5 ± 0.3 | 0.3 ± 0.3 | 3.0 ± 1.2 | | 4.3 ± 1.9 | 10 ± 3.2 | 5.8 ± 2.4 | 3.3 ± 1.1 | 1.7 ± 0.7 |

^1^Total number of individuals recorded.  ^2^Including *L. geniculata, L. moselyi* and early instars identified to genus.
