# Supplementary material for: Characterization of Macroinvertebrate Communities in the Hyporheic Zone of River Ecosystems Reflects the Pump-Sampling Technique Used
Source: PLoS One. 2016 Oct 10;11(10):e0164372. doi: 10.1371/journal.pone.0164372 (PMC5056715; doi:10.1371/journal.pone.0164372)
Supplement: S2 Table — One-way ANOVA tests were conducted where technique*stream/site interactions were significant; df = 1. ‘Common’ taxa are defined in the text. Coarse-grained and fine-grained sites are distinguished for the Albarine and the Bienne due to differences in assemblage composition between sites. Taxa are listed in order of decreasing abundance. Further taxonomic information is given in S3 and S4 Tables. (DOCX) [file pone.0164372.s002.docx]

**S2 Table. Results of two-way ANOVAs to determine differences in macroinvertebrate abundance, taxa richness and the abundance of common taxa between Bou-Rouch (BR) and vacuum pump (VP) sampling techniques used across streams/sites in (a) the UK and (b) France.** One-way ANOVA tests were conducted where technique*stream/site interactions were significant; df = 1. ‘Common’ taxa are defined in the text. Coarse-grained and fine-grained sites are distinguished for the Albarine and the Bienne due to differences in assemblage composition between sites. Taxa are listed in order of decreasing abundance. Further taxonomic information is given in Tables S3-S4.

|  | **ANOVA: between-technique differences** | | | **Technique*stream interaction** | | | | **One-way ANOVA** | | | | | | | | | | | | |
| --- | --- | --- | --- | --- | --- | --- | --- | --- | --- | --- | --- | --- | --- | --- | --- | --- | --- | --- | --- | --- |
|  |  |  |  |  |  |  |  | **Ashop** | | | | | **Black Brook** | | | | | **Lathkill** | | |
| **(a) UK** | ***F*** | **df** | ***P*** | ***F*** | **df** | ***P*** | | ***F*** | | ***P*** | | | ***F*** | | | ***P*** | | ***F*** | | ***P*** |
| **Total abundance** | 47.05 | 1 | <0.001 | 1.58 | 2 | | 0.134 |  | |  | | |  | | |  | |  | |  |
| **Taxa richness** | 26.19 | 1 | <0.001 | 2.06 | 2 | | 0.141 |  | |  | | |  | | |  | |  | |  |
| **Oligochaeta** | 42.66 | 1 | <0.001 | 13.11 | 2 | | <0.001 | 0.62 | | 0.446 | | | 14.43 | | | 0.002 | | 2.54 | | 0.142 |
| **Chironomidae** | 23.95 | 1 | <0.001 | 0.68 | 2 | | 0.510 |  | |  | | |  | | |  | |  | |  |
| ***Leuctra* spp.^2^** | 29.19 | 1 | <0.001 | - | - | | - |  | |  | | |  | | |  | |  | |  |
| ***G. pulex*** | 23.95 | 1 | 0.001 | 0.10 | 1 | | 0.919 |  | |  | | |  | | |  | |  | |  |
|  |  |  |  |  |  | |  | **One-way ANOVA** | | | | | | | | | | | | |
|  |  |  |  |  |  |  |  | **Ain** | | | **Albarine-C** | | | **Albarine-F** | | | **Bienne-C** | | **Bienne-F** | |
| **(b) France** | ***F*** | **df** | ***P*** | ***F*** | **df** | | ***P*** | ***F*** | ***P*** | | ***F*** | ***P*** | | ***F*** | ***P*** | | ***F*** | ***P*** | ***F*** | ***P*** |
| **Total abundance** | 49.73 | 1 | 0.001 | 5.88 | 4 | | 0.001 | 37.8 | <0.001 | | 22.50 | 0.003 | | 9.88 | 0.020 | | 0.38 | 0.560 | 4.68 | 0.074 |
| **Taxa richness** | 37.43 | 1 | <0.001 | 2.39 | 4 | | 0.068 |  |  | |  |  | |  |  | |  |  |  |  |
| **Orthocladiinae** | 44.04 | 1 | <0.001 | 3.04 | 4 | | 0.029 | 19.85 | 0.001 | | 19.34 | 0.005 | | 15.74 | 0.007 | | 2.42 | 0.171 | 3.26 | 0.121 |
| **Oligochaeta** | 22.76 | 1 | <0.001 | 2.14 | 4 | | 0.094 |  |  | |  |  | |  |  | |  |  |  |  |
| ***N. casparyi*** | 53.51 | 1 | <0.001 | 32.20 | 4 | | <0.001 | - | - | | 134.6 | <0.001 | | 1.10 | 0.334 | | - | - | 0.429 | 0.537 |
| ***Esolus* sp.** | 30.04 | 1 | <0.001 | 4.93 | 4 | | 0.003 | 62.95 | <0.001 | | 3.50 | 0.110 | | 6.38 | 0.045 | | 0.021 | 0.890 | 8.63 | 0.026 |
| **Valvatidae** | 14.00 | 1 | 0.001 | 3.52 | 4 | | 0.015 | 1.547 | 0.234 | | 7.82 | 0.031 | | 2.07 | 0.200 | | 3.00 | 0.134 | 5.29 | 0.61 |
| **Hydrachnida** | 5.83 | 1 | 0.021 | 2.99 | 2 | | 0.031 | 15.09 | 0.002 | | 5.42 | 0.59 | | 0.01 | 0.973 | | 0.907 | 0.378 | 2.43 | 0.170 |
| **Chironominae** | 23.50 | 1 | <0.001 | 0.93 | 4 | | 0.456 |  |  | |  |  | |  |  | |  |  |  |  |
| ***G. fossarum*** | 33.48 | 1 | <0.001 | 7.42 | 2 | | <0.001 | 37.97 | <0.001 | | 2.72 | 0.150 | | 157.7 | <0.001 | | 0.75 | 0.420 | 0 | 0.986 |
| ***Leuctra fusca*** | 0.39 | 1 | 0.535 | 0.07 | 4 | | 0.990 |  |  | |  |  | |  |  | |  |  |  |  |
| ***Limnius* sp.** | 1.44 | 1 | 0.238 | 0.92 | 4 | | 0.461 |  |  | |  |  | |  |  | |  |  |  |  |
